# Dolutegravir-associated resistance mutations after first-line treatment failure in Brazil

**DOI:** 10.1186/s12879-023-08288-8

**Published:** 2023-05-24

**Authors:** Ricardo Sobhie Diaz, James R. Hunter, Michelle Camargo, Danilo Dias, Juliana Galinskas, Isabela Nassar, Isaac Barbosa de Lima, Debora Bellini Caldeira, Maria Cecilia Sucupira, Mauro Schechter

**Affiliations:** 1 Federal University of São Paulo, São Paulo, Brazil; 2 Federal University of Rio de Janeiro, Rio de Janeiro, Brazil

**Keywords:** Dolutegravir, Resistance associated mutations, Transmitted drug resistance, Virologic failure, Brazil

## Abstract

**Background:**

Since January 2017, the recommended first-line antiretroviral regimen in Brazil is the fixed-dose combination of tenofovir plus lamivudine with dolutegravir (TL + D). According to the literature, integrase resistance-associated mutations (INRAMs) are rarely found upon virologic failure to first-line dolutegravir plus two nucleoside reverse transcriptase inhibitors. We evaluated the HIV antiretroviral genotypic resistance profile of patients referred for genotyping in the public health system who failed first-line TL + D after at least six months of therapy on or before December 31, 2018.

**Methods:**

HIV Sanger sequences of the pol gene were generated from plasma of patients with confirmed virologic failure to first-line TL + D in the Brazilian public health system before December 31, 2018.

**Results:**

One hundred thirteen individuals were included in the analysis. Major INRAMs were detected in seven patients (6.19%), four with R263K, one with G118R, one with E138A, and one with G140R. Four patients with major INRAMs also had the K70E and M184V mutations in the RT gene. Sixteen (14.2%) additional individuals presented minor INRAMs, and five (4,42%) patients had both major and minor INRAMS. Thirteen (11.5%) patients also presented mutations in the RT gene selected by tenofovir and lamivudine, including four with both the K70E and M184V mutations and four with only M184V. The integrase mutations L101I and T124A, which are in the in vitro pathway for integrase inhibitor resistance, were found in 48 and 19 patients, respectively. Mutations not related to TL + D, thus probable transmitted resistance mutations (TDR), were present in 28 patients (24.8%): 25 (22.1%) to nucleoside reverse transcriptase inhibitors, 19 (16.8%) to non-nucleoside reverse transcriptase inhibitors, and 6 (5.31%) to protease inhibitors.

**Conclusions:**

In marked contrast to previous reports, we report a relatively high frequency of INRAMs among selected patients failing first-line TL + D in the public health system in Brazil. Possible reasons for this discrepancy include delays in detecting virologic failure, patients inadvertently on dolutegravir monotherapy, TDR, and/or infecting subtype.

## Background

Antiretroviral regimens containing integrase strand-transfer inhibitors (INSTIs) have been shown to have greater efficacy, safety, and fewer drug-drug interactions for the initial treatment of HIV infection than regimens containing non-nucleoside reverse transcriptase inhibitors (NNRTI) or protease inhibitors (PI) [1–6]. Accordingly, all major HIV treatment guidelines presently recommend the inclusion of INSTIs as part of first-line regimens.

In 1991, Brazil became the first middle-income country to provide free and universal access to antiretroviral therapy (ART) to all people living with HIV (PLWH) who qualify for treatment. Its Ministry of Health (MoH) periodically updates guidelines developed by an independent advisory panel. Since 2014, these guidelines have recommended that all PLWH be started on therapy regardless of symptoms or CD4 + T cell count [7].

Starting in January 2017, the recommended first-line regimen in Brazil is the fixed-dose combination of generic tenofovir 300 mg plus lamivudine 300 mg, associated with 50 mg of the INSTI dolutegravir (DTG), a regimen known as TL + D. DTG is a second-generation INSTI with a high genetic barrier to resistance and few drug-drug interactions [8, 9]. In addition, in clinical trials and large-scale public health rollout programs, mutations in the integrase gene associated with antiretroviral resistance have rarely been described after virologic failure to first-line DTG-containing regimens [2, 9, 10].

Although viral load monitoring is performed at the attending physician's discretion, measurements with intervals of less than six months are necessary for patients to continue to receive antiretrovirals through the Brazilian public health system [7].

In 2001 the Brazil National AIDS Program created a national network for genotyping (RENAGENO), which developed methods and guidelines to standardize resistance testing in the public health system [11]. Since January 2017, all resistance testing in the public health system in Brazil has been carried out in a central laboratory located in São Paulo (Laboratório Centro de Genomas). Pre-treatment genotyping in Brazil is not usually allowed through the public system, except for children up to 12 years of age, individuals with documented recent seroconversion, individuals with an HIV-infected sexual partner on ART, pregnant women, and patients with tuberculosis. Women of childbearing potential who are not using contraceptives are also entitled to pre-treatment genotyping.

According to national guidelines, all patients who had at least six months of ART and confirmed virologic failure (a second detectable viral load at least four weeks after the first one) are entitled to HIV genotypic resistance testing.

As of December 2021, approximately 410,000 individuals were using DTG in Brazil, including those who had started DTG as first-line therapy, those who had switched to a DTG-containing regimen without having experienced prior virologic failures, or those who were using DTG after one or more virologic failures (Brazil Ministry of Health) [7]. At the time, it was estimated that after six months of starting first-line DTG, 91% of patients had viral loads < 50 copies/mL [12].

Here we present the results of 113 patients who failed the first-line TL + D regimen after at least six months of therapy and for whom resistance tests were requested to the national reference laboratory on or before December 31, 2018.

## Methods

This was a retrospective evaluation of genotypic resistance profiles of individuals with confirmed virologic failure after at least six months of first-line TL + D for whom resistance tests were requested to the Brazilian national reference laboratory before December 31, 2018. Confirmed virologic failure was defined as two successive detectable viral loads at least four weeks apart. Plasma samples from the second blood draw were evaluated. Sanger sequences of the integrase (IN), protease (PR), and reverse transcriptase (RT) regions of the *pol* gene were generated as previously described [13, 14]. Resistance mutations were classified according to the 2019 IAS-USA updated drug resistance mutations list [15]. Subtype assignment was confirmed by phylogenetic analysis. The HIV nucleotide sequences were submitted to the GeneBank, Accession numbers (pending).

This study was approved by the local Ethical Review Board (# 19220719.7.0000.5505). The Brazilian MoH granted access to the national databases for PLWH (protocol # 25820009249201976, December 10, 2019). However, the Brazilian MoH had no role in the analysis or the interpretation of the data, and the views presented in this article are the entire responsibility of the authors.

The Brazilian MoH has three large national databases for PLWH. The first, SISGENO, has the genotype results from all patients tested since the creation of RENAGENO in 2001, including FASTA sequence files, corresponding plasma HIV RNA viral loads, and CD4 + T cell counts results, all of which are automatically entered into the database. It also has information on treatment regimens being used by these patients.

The second database, SISCEL, includes all lymphocyte CD4 + T cell and CD8 + T cell counts and viral loads performed in the Brazilian public health system since 1996. These data are automatically entered into the database through interfaces between the equipment performing the laboratory tests and the database.

The third database is named SICLOM. The pharmacist responsible for each drug dispensation loads the data at the local dispensary. The software possesses several internal controls that prevent the distribution of drugs and/or regimens that are not in accordance with the national ART guidelines. For example, a patient being included for the first time in the system will only be allowed to receive TL + D. Special authorizations are necessary to dispense a different regimen, and justifications and names of the persons who authorized the dispensation are clearly marked in the system.

To ensure that only patients failing first-line TL + D after six months of therapy were included in the present analysis, several precautions were taken. We first identified all first-line TL + D treatment failures in the RENAGENO database that occurred after July 2017. Next, we checked the SICLOM database to verify if each identified individual had not previously received antiretroviral drugs. In some cities, because data entry in the SICLOM database only became fully automated in 2011, we excluded patients from these locations from the analysis. If there was evidence of drastic declines in plasma viral load or significant CD4 + T cell count increases in the SISCEL database before initiation of TL + D, that patient was also excluded from the analysis as these changes might represent undocumented exposure to antiretroviral drugs.

Data cleaning, organization, description, and statistical analysis were performed using the R Language and Environment for Statistical Computing [16] and its tidyverse data handling packages [17]. The non-parametric Wilcoxon Rank Sum test analyzed differences in viral loads between groups.

## Results

From July 2017 to December 2018, 113 antiretroviral naïve individuals who had started first-line TL + D had confirmed virologic failure after at least six months of treatment. Those who conformed to the inclusion criteria were identified and included in the present study. This series of patients includes all PLWH who failed first-line TL + D during the study period and for whom the attending physician requested a genotypic resistance test through the Brazilian public health system.

The demographic virologic and immunologic characteristics of the patients and the mean HIV viral loads and CD4 + T cell counts at baseline, and upon treatment failure are presented in Tables 1 and 2. Of the 113 patients, 100 had genders reported. Seventy-three (73%) of these were male, and 27 (27%) were female. The mean age of the patients was 36.1 years (sd = 10.77 years). The mean viral load immediately before treatment initiation (baseline) was 5.67 log_10_, whereas the mean CD4 + T cell count was 323.2. At virologic failure, the mean viral load was 5.13 log_10_, whereas the mean CD4 + T cell count was 365.9 (Tables 1 and 2).

**Table 1:** Mean and standard deviation of demographic characteristics, Viral loads and CD4 + T cell counts at baseline and upon virologic failure according to the HIV resistance profile

	**N**	**Age (years)**^a^	**Males n(%)**	**Females n(%)**	**Viral Load at virologic failure**^a^	**CD4 at virologic failure**^a^	**Baseline Viral Load**^a^	**Baseline CD4**^a^
Wild Type HIV	65	35.7(10.27)	42(73.68)	15(26.32)	4.91(5.263)	438(332.5)	5.66(6.112)	391(359)
TL + D RAMS	2	34.0(4.243)	2(100.00)	0(0.00)	5.35(5.413)	53.5(30.41)	6.12(6.112)	45.5(55.86)
TL RAMS	6	37.2(15.79)	3(50.00)	3(50.00)	5.44(5.662)	175(172.6)	5.87(6.029)	161(170.8)
Major INRAMS	7	37.9(14.35)	5(71.43)	2(28.57)	5.01(5.145)	238(218.4)	5.73(5.935)	184(211.2)
Minor INRAMS	18	33.6(10.58)	12(75.00)	4(25.00	5.17(5.411)	281(237.9)	5.77(5.84)	179(178.85)
NRTI RAMS	15	35.1(11.43)	10(71.43)	4(28.57)	5.50(5.853)	221(188.7)	5.90(6.015)	207(193.8)
NNRTI RAMS	16	35.5(12.29)	9(69.23)	4(30.77)	5.17(5.595)	356(200.6)	5.35(5.686)	304(149.8)
PI RAMS	6	38.8(10.43)	4(80.00)	1(20.00)	4.93(5.077)	216(176.8)	5.28(5.286)	212(158.2)
Total	113^b^	36.1(10.77)	73(73.00)	27(27.00)	5.13(5.55)	365.9(294.1)	5.67(6.034)	323.2(305.6)

The total n of 113 is less than the sum of the n’s in the categories as there were many cases that had multiple categories of RAMs

*TL* Tenofovir/lamivudine, *D* Dolutegravir, *RAM* Resistance-associated mutations, *INRAM* Integrase resistance-associated mutations, *NRTI* Nucleoside reverse transcriptase inhibitor, *NNRTI* Non-nucleoside reverse transcriptase inhibitor, *PI* Protease inhibitors

^a^Mean(std. dev.)

^b^one sample failed in the RT and protease PCR amplification

**Table 2:** Mean and standard deviation of demographic characteristics, Viral loads and CD4 + T cell counts at baseline and upon virologic failure according to the HIV subtype

	**N**	**Age (years)**^a^	**Males n(%)**	**Females n(%)**	**Viral Load at virologic failure**^a^	**CD4 at virologic failure**^a^	**Baseline Viral Load**^a^	**Baseline CD4**^a^
Clade B	80	36.8(10.23)	56(75.68)	18(24.32)	5.19(5.602)	362.3(322.3)	5.65(6.064)	306.4(300.0)
Non-Clade B	27	34.2(11.96)	14(66.67)	7(33.33)	4.92(5.301)	386.5(210.4)	5.81(5.994)	361.8(266.7)
Recombinant	6	34.4(14.72)	3(60)	2(40)	4.93(5.139)	327.2(185.4)	4.97(5.123)	443.2(465.5)
Total	113^b^	36.1(10.77)	73(73)	27(27)	5.13(5.55)	365.9(294.1)	5.67(6.034)	323.2(305.6)

Totals for gender in the categories were subject to a number of missing cases

^a^Mean(std. dev.)

^b^one sample failed in the RT and protease PCR amplification

One hundred seven of the 113 participants had HIV subtypes recorded for the protease and reverse transcriptase genes (six samples failed to generate protease and reverse transcriptase PCR products for sequencing). Among samples that failed to generate protease and reverse transcriptase PCR products, four were classified as subtype B, one as C, and one as F, according to the integrase profile. Of these, 77 (68.1%) were of subtype B, 12 (10.6%) of subtype C, three recombinant B/C (2,6%), 12 (10.6%%) were of subtype F and three recombinant B/F (2,6%). At the integrase gene, all 113 participants had subtypes recorded. Of these, 80 (70.8%) were of subtype B, 12 (10.6%) were of subtype C, four recombinant B/C (3.5%), 15 (13.3%) of subtype F, and two were recombinant B/F (1.8%).

Integrase resistance-associated mutations were detected in 25 (22.1%) participants. Major INRAMs were detected in seven participants (6.19%): four with the DTG-specific mutation R263K, of whom three were clade B, and one clade C in the integrase gene. Of the other three participants with major INRAMs, one had the G118R mutation (clade C), one carried the E138A mutation (clade B), and one harbored the G140R mutation (BC recombinant virus). Two patients with the R263K INRAM also had both the K70E and M184V mutations in the RT gene.

Eighteen (15.9%) additional individuals presented with minor INRAMs, 15 with a single mutation, and three with two mutations (M50I + L74I and M50I + G193E). It should be noted that five patients had both major and minor INRAMS, which were the association of R263K/R with M50I/T and L101I/V (sample 11, Table 4), G140R with G163R (sample 15), E138A with V151A (sample 68), R263K with E157Q (sample 73), R263K/R with L101I and G149A/G (sample 90). Table 3 summarizes the INRAM totals across the sample, while Table 4 shows the mutations for all participants.

**Table 3:** Mean HIV viral loads and mean CD4 + T cell counts according to the prevalence of Integrase Resistance Associated Mutations (INRAMS)

Mutations Profile	Number	Log_10_ VL	CD4^+^
Major INRAMS	7	5.01	237.71
G118R	1	4.60	146.00
E138A	1	4.73	29.00
R263K	4	5.16	236.25
G140R	1	4.62	544.00
Minor INRAMS (all)	18	5.17	281.07
Major plus Minor INRAMs	5	5.15	278.06
Minor INRAMS (additional)	13	5.22	306.30
Single Minor INRAM	15	5.22	279.92
More than 1 Minor INRAM	3	4,77	288,5
L101I & T124A Pathway^α^			
L101I	46	5.17	458.16
T124A	22	5.03	281.35
Both	11	5.04	254.50
Either	57	5.14	421.76

*VL* HIV RNA viral load, *CD4* CD4 positive T cell counts

^α^The L101I and T124A integrase emerge in vitro as a pathway to dolutegravir resistance

**Table 4:**
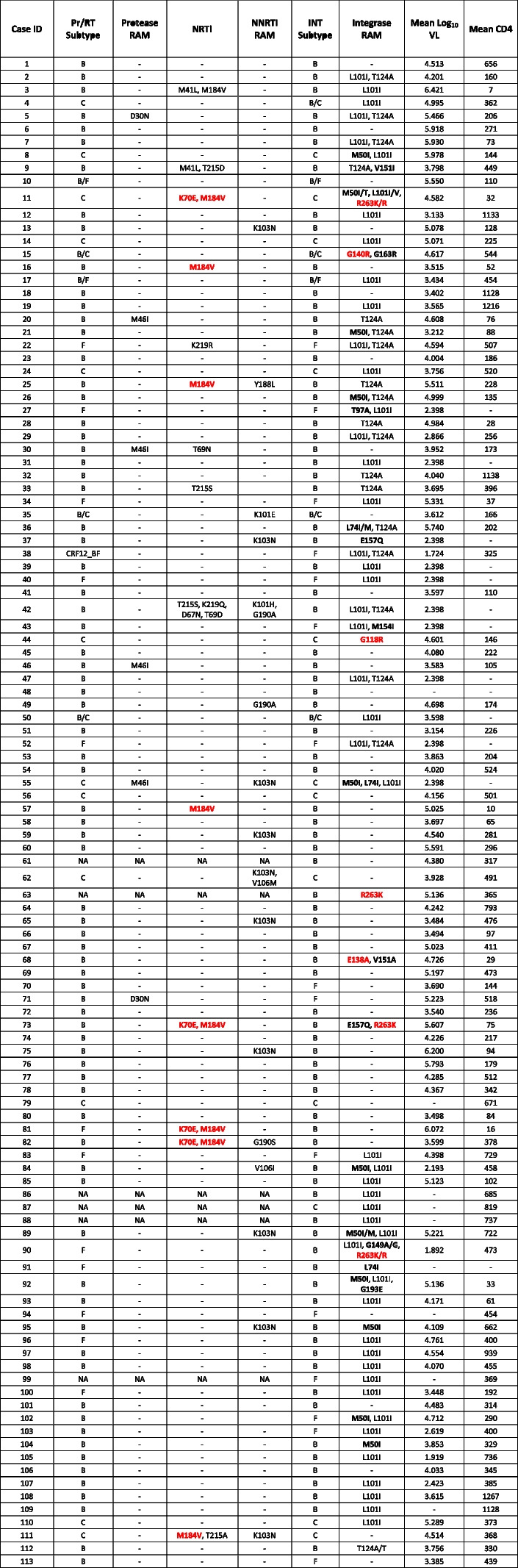
HIV subtype profile, Resistance Associated Mutations (RAM), HIV RNA viral loads (VL), and CD4 positive T cell counts (CD4) for All Cases. RAM associated with tenofovir, lamivudine, and major Integrase RAMs (https://hivdb.stanford.edu/dr-summary/resistance-notes/INSTI/) are highlighted in red. Minor INRAMs are marked in bold. *Pr/RT* Protease and reverse transcriptase regions of *pol* gene, *INT* Integrase region of the *pol* gene, *NA* Not available due to a negative, *PCR* Dashes indicate absence of RAMs

The integrase mutations L101I and T124A, which are in the in vitro pathway for integrase inhibitor resistance [18, 19], were found in 46 (40.7%) and 22 (19.5%) participants, respectively. These mutations were jointly found in 11 individuals (9.7%).

Thirteen (11.5%) participants presented with tenofovir and/or lamivudine resistance mutations in the RT gene, including four (3.5%) with both the K70E and M184V mutations. The other nine participants presented only the M184V mutation. One patient with the M184V mutation also had the thymidine analog mutation M41L mutation, which is not selected by either tenofovir or lamivudine.

Drug resistance mutations (DRMs) not associated with resistance to tenofovir, lamivudine, or DTG, thus possibly transmitted drug resistance mutations, were detected in 28 participants (24.8%). These include 15 participants (13.3%) with DRMs to nucleoside reverse transcriptase inhibitors (NRTIs), 16 (14.2%) to NNRTIs, and 6 (5.3%) to PIs (Table 4). In addition, T215 revertants, considered a hallmark of TDR, were found in four samples, including T215D (sample 9, Table 4), T215S (samples 33 and 42), and T 215A (sample 111).

The mean viral load upon TL + D failure was 5.14 log_10_ copies/mL for the 88 individuals with no INRAMs, compared to 5.15 log_10_ copies/mL among individuals with any INRAM (25 individuals, Table 4). The difference between the presence or absence of an integrase INRAM according to the HIV viral load is not statistically significant (Wilcoxon rank-sum test, W = 650, *p* = 0.331). The mean viral load among the seven individuals with major INRAMs was 5.17 log_10_ copies/mL. The difference between this value and those participants with no major INRAM is also not statistically significant (Wilcoxon rank-sum test, W = 440.5, *p* = 0.192).

The mean CD4 + T cell count among individuals upon TL + D failure with no INRAMs was 383.06 compared to 287.56 among individuals with any INRAM. The mean CD4 + T cell count was 237.71 for the seven individuals with major INRAMs. These differences are not statistically significant (Wilcoxon rank-sum test, W = 236, *p* = 0.232). Table 4 depicts the characteristics of participants with TL + D virologic failure and INRAMs.

There was no association between the prevalence of resistance of all three analyzed genomic regions and gender, age, geographic region, or infecting HIV subtype.

## Discussion

Notably, in the registration clinical trials involving treatment naïve patients who started on the combination of DTG plus two NRTIs, no known INRAMs were detected in patients with protocol-defined virologic failure [1, 3–5]. Similarly, the combination of DTG and two NRTIs showed high efficacy in treatment-experienced, INSTI naïve patients and a very low risk of developing acquired drug resistance in case of virologic failure [20]. Additionally, there is an extremely limited number of anecdotal reports of the development of INRAMs in patients failing DTG-containing first-line regimens in clinical practice [9].

It is not entirely understood why drug resistance has been so rarely reported in the event of virologic failure during first-line treatment with DTG plus two NRTIs. Near-complete adherence or non-adherence will likely result in complete viral suppression or the rebound of wild-type virus, respectively. Sanger sequencing, the most widely used method, will only detect resistant variants present in ≥ 20% of the viral population [21, 22]. On the other hand, partial or intermittent adherence may result in minority resistant variants representing less than 20% of the viral population. In this situation, resistant viruses can only be detected by next-generation sequencing techniques [22], which are not routinely used. Additionally, clinical trials may not predict the effectiveness of DTG-based regimens in real life since the presence of resistance mutations to any of the components of the study regimens usually is an exclusion criterion. Viral load is also frequently monitored, leading to prompt switching of treatment regimen in case of virologic failure. Both of these conditions do not occur typically in clinical practice, particularly in resource-limited settings (RLS). Early switching of treatment regimen may not allow minority variants to outgrow wild-type viruses and thus be detected by routine resistance testing. Therefore, it is unclear if the lack of acquired drug resistance to DTG observed in clinical trials can be directly extrapolated to clinical practice, particularly to settings where pre-treatment resistance testing and frequent viral load monitoring are not routinely performed.

Clinical trials evaluating the efficacy of DTG-based regimens were mainly performed in high-income countries where most PLWH are infected with HIV-1 subtype B [23, 24]. Additionally, most in vitro characterizations of DTG resistance-associated mutations have been performed exclusively for HIV subtype B virus [25]. Worldwide, subtype B is responsible for only approximately 10% of the infections. Subtype C, which is rare in high-income countries, represents 50% of the infections in RLS. In Brazil, the subtype distribution varies markedly by geographic region. Overall, approximately 70% of individuals are infected with subtype B, which co-circulates with subtype F and with BF recombinant forms (approximately 20%, mostly in the Northeast Region) and subtype C and C recombinant forms (approximately 10%, mostly in the south of the country) [26]. The BF recombinant forms in the integrase gene originate from subtypes B or F or a combination of both [27]. For DTG and other INSTIs, differences in susceptibilities and mutational patterns of resistant viruses across HIV subtypes have been observed in vitro [25, 28, 29]*.* In addition, poorer virologic responses have been reported for subtype F compared to subtype B for individuals treated with an INSTI as first-line therapy [18, 19]. The importance of these subtype-specific differences in determining the risk for virologic failure and acquired DTG resistance remains to be determined.

In marked contrast with clinical trials [2, 9, 10], major INRAMs were found in 6.19% of this sample of 113 patients who failed first-line TL + D in the public health system in Brazil after at least six months of treatment and for whom resistance tests were requested to the national reference laboratory by December 31, 2018. Nonetheless, it should be noted that in clinical trials, the emergence of RAMs after first line virologic failure using NNRTIs such as efavirenz is much higher, and, in general, up to 50% of virologically failing patients harbor NNRTI RAMs, half of these also harboring the M184V mutation [30].

In the DOMONO study, in which 95 patients were treated for 24 weeks with DTG monotherapy, 8.4% had virologic failure, and 3.2% had INRAMs [31]. It should also be speculated that the genetic barrier of TL + D is higher than dual therapy using L + D, since one patient of 716 using L + D in the GEMINI study developed HIV harboring M184V plus R263R/K upon virologic failure compared to none of the 717 patients using TL + D [32]. Similarly, one patient of 120 from ACTG-A5353 also developed M184V and R263K mutation [33]. Although our data do not allow us to speculate about the emergence of resistance of dual therapy using DTG and one NRTI in real life, it is conceivable that these schemes may incur higher risks of selection of RAM than what was seen with TL + D here.

Although the subtype distribution of the 113 patients failing first-line TL + D mirrored the distribution of subtypes in Brazil, the small sample size does not allow us to reach any conclusions relative to their role, if any, in the development of INSTI-resistance mutations.

Interestingly, the prevalence of two integrase polymorphisms, L101I and T124A, was higher than usually found among integrase-naïve Brazilian patients, especially the T124A mutation that had previously been found in only 12% of patients [14]. These two mutations may be considered pathways to resistance to DTG since in vitro T124A emerges on day 14 of co-culture and L101I after 70 days [34]. The presence of L101I and T124A, in association with the 153F mutation (not present in this sample) is associated with a modest decrease in susceptibility to DTG (fold change of 1.9). We recognize that it is unclear the effect of two mutations in the susceptibility of HIV to DTG and that these mutations are not included in algorithms for resistance interpretation of INSTIs. However, it is conceivable that L101I and/or T124A could have been selected in vivo by patients failing TL + D. Nonetheless, phenotypic resistance tests in the set of samples harboring L101I and or T124A mutations in patients failing TL + D regimens may elucidate the role of these substitutions in the decrease of DTG susceptibility.

It has been demonstrated that specific DTG RAMs, such as the mutations at integrase codon 263, lead to strains with extremely low fitness [35] and low viral loads upon virologic failure [20]. This is not what was observed in the present real-life study, where patients failing first-line TL + D with the R263K INRAM had high viral loads. Only one patient harboring HIV with the R263K mutation presented a low viral load (patient ID 90, viral load of 1.9 log_10_). Other PLWH harboring HIV with this mutation showed viral loads of 4.6 log_10_ (patient 11, Table 4), 5.1 log_10_ (patient 63, the only patient carrying a single INRAM), and 5.6 log_10_ (patient 73). It has been shown that when drug-resistance mutations accumulate over time, there is a trend for viral fitness to be restored and for the viral load to increase [36]. It is possible that HIV genomic regions related to DTG resistance, such as the 3´PPT [37] that we have not studied, might contribute to restoring HIV fitness and to increasing viral load upon virologic failure. Mutations in the HIV-1 3’PPT among patients failing dolutegravir in Brazil have been detected in 6 of 51 patients, one of them also harboring the R263K mutation [38]. Interestingly, the allosteric integrase inhibitors which interact with the non-catalytic site in HIV integrase may act in three distinct steps of the HIV replication cycle. Besides blocking the cDNA integration into human chromatin, there is also a potent effect in the final steps of the viral replicative cycle, preventing virus assembly [39], and a blockage of reverse transcription in the next cycle of replication [40]. Regarding the unique reverse transcriptase inhibition, one can hypothesize that the K70E mutation found in four patients might be a landmark of a TL + D resistance pathway. The tenofovir K70E mutation, which was found in four patients, is exceedingly rare in Brazil and elsewhere compared to the K65R mutation [36, 41].

Of note, one patient infected by a recombinant B/C strain presented the recently described G140R mutation, associated G163R (Table 4). The G140R mutation was first described in macaques receiving long-acting cabotegravir for pre-exposure prophylaxis (PrEP) [42] and in patients failing treatment with the combination of cabotegravir and rilpivirine and harboring viruses of clades A6/A1 [43]. G140R is considered an infrequent mutation, reported in one PLWH failing cabotegravir [44], which led to a 6.7-fold reduction in cabotegravir susceptibility [43]. It is, therefore, conceivable that this mutation could also be selected by DTG upon virologic failure, and the confirmation of phenotypic resistance to INSTIs on this isolate would be of importance.

In the present study, TDR was present in a quarter of the participants who failed first-line TL + D. This is significantly higher than previously reported in ART-naïve patients [26, 27, 45]. The evidence for the TDR is the presence of mutations not selected by TL + D. As seen in Table 4, the protease mutations D30N (nelfinavir, two isolates) and M46I (indinavir, four isolates) indicate ancient TDR chains since these two PIs have not been available used in Brazil for over a decade. The same rationale applies to the T69D mutation selected by ddI, a drug that has not been available in Brazil for a long time. Thymidine analog mutations were detected in many isolates, including AZT revertants such as T215D, S, or A, a hallmark of TDR. Also, mutations related to efavirenz and nevirapine, which are, in general, the more prevalent TDR mutations, were frequently detected (Table 4). It needs to be mentioned that in Brazilian non-B isolates, the prevalence of TDR can be underestimated since the genotypic correlates of phenotypic resistance may not be straightforward [46].

In some of these cases, it is possible that TDR to other components of the regimen may play a role in the failures of first-line regimens containing DTG. Since many mutations associated with drug resistance may persist over time [47], pre-treatment genotyping can be a highly sensitive method to detect TDR. However, the prevalence of some transmitted RAMs in treatment-naïve patients may vary over time and exist as minority populations that can only be detected by next-generation sequencing (NGS). In fact, there is evidence that TDR present in minority HIV-1 populations detected by NGS may impact virologic response to first-line ART regimens if adherence to treatment is poor [48]. Therefore, one can speculate that TDR can contribute to the failure to first-line treatment with TL + D, particularly in the setting of poor adherence. TL + D is a two-pill regimen. There are studies that indicate that adherence to single-pill regimens is higher than to ones using two or more pills [49]. It is conceivable that some patients elect to take only one of the pills (DTG), which effectively places them on monotherapy and therefore favors the selection of INRAMs. Interestingly, the M184V mutation was present in six isolates (Table 4, case ID # 11, 16, 25, 73, 57, 81). It is not possible to determine whether these were selected or transmitted mutations. Of note, in isolate ID #111 on Table 4, the revertant mutation T215A was also present. However, although M184V is one of the most frequent emerging mutations in virologic failure, it is a rare mutation in naïve patients, probably because it is one of the few mutations that revert over time without the selective pressure of antiretrovirals [47]. Nonetheless, this was an essential mutation in this casuist, reflecting regimen failure with or without DTG resistance.

In Brazil, the only INSTI available before December 2017 was raltegravir, and its use from January 2009 to 2017 was limited to salvage therapy for patients with documented resistance to PIs. It was usually used in combination with one or more NRTIs, a boosted PI, with or without etravirine and/or maraviroc [7]. Therefore, TDR that includes INRAMs selected by raltegravir are to be expected. In fact, mutations and polymorphisms associated with raltegravir resistance, such as E138A, L74I/M, G163R, V151A/I, T97A, and E157Q, were present in some patients failing TL + D [13]. Although pre-treatment genotype was not performed in this group of patients, it cannot be ruled out that transmitted raltegravir-associated resistance mutations and polymorphisms might have played a role in virologic failures. Exposure to DTG leads to a further selection of DTG-associated DRMs, such as R263K or G118R.

The prevalence of viremia above 50 copies/mL in clinical trials using DTG-based regimens for first line treatment is between 1 and 3% at weeks 24 or 48 [1–5]. In contrast, in the “real world” setting in Brazil, as much as 9% of PLWH starting TL + D present viral loads > 50 copies/mL at weeks 24 or 48 of treatment [12]. In 2017, 70,250 PLWH started ART in Brazil, 77% with TL + D. In 2018, 68,626 started ART, 86% with TL + D [7]. Assuming that 9% of them would have failed virologically after 6 months of therapy, over 12,000 patients on TL + D would have been entitled to have genotype testing. However, we were able to identify only 113 genotype tests that met the study criteria. It should be noted that the central laboratory performed at the time an average of 1,500 genotype tests per month. It is unclear why so few tests were from patients failing first-line TL + D, but it is conceivable that the perception among attending physicians that in most such cases salvage therapy would be successful without the need of a resistance test played an important role. Therefore, it is also conceivable that genotype testing was requested only for the most hard-to-treat, less adherent patients failing TL + D. This, in turn, would represent a very important selection bias, possibly overestimating the true frequency of antiretroviral resistance among all PLWH failing first-line TL + D. Still, the majority of PLWH failing TL + D as a first-line regimen in real-world settings will harbor HIV without RAMs. Therefore, patients experiencing virological failure on a first line DTG-containing regimen should not be empirically hanged to a second-line regimen. Instead, adherence should be reinforced, and genotypic resistance testing should be considered.

## Conclusions

In marked contrast to what has been reported both in clinical trials and in clinical practice, we report a relatively high frequency of INRAMs among patients failing first-line therapy with a DTG associated regimen and two NRTIs in the public health system in Brazil. Possible reasons for this discrepancy include delays in requesting genotype testing, monotherapy with DTG by some patients due to selective inadequate adherence to the pill containing the NRTIs, TDR to other components of the regimen or to raltegravir, selection of mutations that are in the pathway to integrase inhibitor resistance, and the infecting subtype. Given their potential impact on global public health policies, these results merit further investigation.

## Data Availability

All data for this study and R scripts that analyze it are available at the OpenScience Foundation Repository Dolutegravir-Associated Resistance Mutations in Brazil (https://doi.org/10.17605/OSF.IO/4QAMZ).

## Abbreviations

INSTI: Integrase strand-transfer inhibitor
NRTI: Nucleoside reverse transcriptase inhibitor
NNRTI: Non-nucleoside reverse transcriptase inhibitor
PI: Protease inhibitor
ART: Antiretroviral therapy
PLWH: People living with HIV
MoH: Ministry of Health
DTG: Dolutegravir
TL + D: Fixed dose combination of Tenofovir and Lamivudine associated with Dolutegravir
RENAGENO: Brazilian national network for genotyping
SISGENO: MoH control system for genotype test
SISCEL: MoH control system for laboratory test of lymphocyte T cell counts (CD4 + /CD8 +) and viral load
SICLOM: MoH control system for HIV drugs
INRAM: Integrase resistance-associated mutation
RLS: Resource-limited settings
PPT: Polypurine tract
NGS: Next-generation sequencing
